# 2-Benzoyl-4-chloro­phenyl benzoate

**DOI:** 10.1107/S1600536813014396

**Published:** 2013-05-31

**Authors:** Bushra Begum A, Mohammed Al-Ghorbani, Suresh Sharma, Vivek K. Gupta, Shaukath Ara Khanum

**Affiliations:** a Department of Chemistry, Yuvaraja’s College, University of Mysore, Mysore 570 005, India; b Post-Graduate Department of Physics and Electronics, University of Jammu, Jammu Tawi 180 006, India

## Abstract

In the title compound, C_20_H_13_ClO_3_, the dihedral angles between the benzoate and the chloro­benzene and benzoyl rings are 68.82 (5) and 53.76 (6)°, respectively, while the dihedral angle between the benzoyl and benzoate rings is 81.17 (5)°. The eight atoms of the benzoyl residue are essentially planar with the exception of the O atom which lies 0.1860 (5) Å out of their mean plane (r.m.s. deviation = 0.97 Å). The nine atoms of benzoate residue are also essentially planar (r.m.s. deviation = 0.20 Å) with the ester O atom showing the greatest deviation [0.407 (12) Å] from their mean plane. In the crystal, mol­ecules are connected into centrosymmetric dimers by pairs of C—H⋯O hydrogen bonds.

## Related literature

For related structures, see: Sieroń *et al.* (2004 ▶); Mahendra *et al.* (2005 ▶); Naveen *et al.* (2006 ▶). For the biological activity of the title compound, see: Belluti *et al.* (2011 ▶); Revesz *et al.* (2004 ▶); Khanum *et al.* (2004 ▶, 2009 ▶, 2010 ▶). For bond-length data, see: Allen *et al.* (1987 ▶).
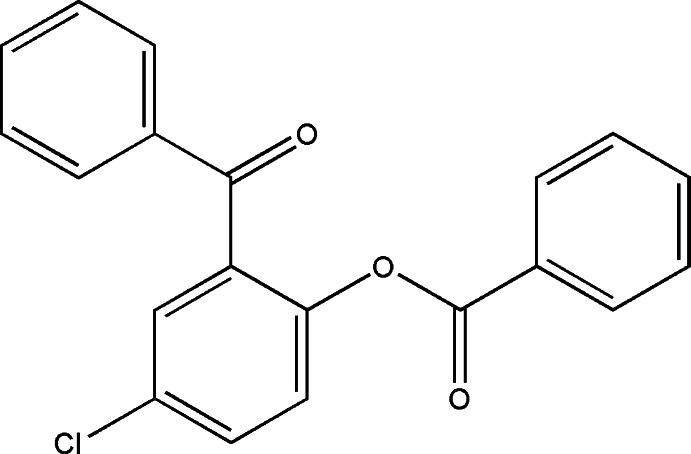


## Experimental

### 

#### Crystal data

C_20_H_13_ClO_3_
*M* *_r_* = 336.75
Triclinic, 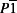

*a* = 9.1934 (2) Å
*b* = 9.8641 (3) Å
*c* = 10.0778 (3) Å
α = 94.033 (2)°
β = 114.207 (2)°
γ = 102.512 (2)°
*V* = 800.64 (4) Å^3^
*Z* = 2
Mo *K*α radiation
μ = 0.25 mm^−1^
*T* = 293 K
0.30 × 0.20 × 0.20 mm

#### Data collection

Oxford Xcalibur Sapphire3 diffractometer
Absorption correction: multi-scan (*CrysAlis RED*; Oxford Diffraction, 2010 ▶) *T*_min_ = 0.871, *T*_max_ = 1.000
18813 measured reflections
3131 independent reflections
2631 reflections with *I* > 2σ(*I*)
*R*_int_ = 0.031

#### Refinement

*R*[*F* ^2^ > 2σ(*F* ^2^)] = 0.035
*wR*(*F* ^2^) = 0.084
*S* = 1.03
3131 reflections
217 parameters
H-atom parameters constrained
Δρ_max_ = 0.20 e Å^−3^
Δρ_min_ = −0.21 e Å^−3^

### 

Data collection: *CrysAlis PRO* (Oxford Diffraction, 2010 ▶); cell refinement: *CrysAlis PRO*; data reduction: *CrysAlis PRO*; program(s) used to solve structure: *SHELXS97* (Sheldrick, 2008 ▶); program(s) used to refine structure: *SHELXL97* (Sheldrick, 2008 ▶); molecular graphics: *ORTEP-3* (Farrugia, 2012 ▶); software used to prepare material for publication: *PLATON* (Spek, 2009 ▶).

## Supplementary Material

Crystal structure: contains datablock(s) I, global. DOI: 10.1107/S1600536813014396/go2090sup1.cif

Structure factors: contains datablock(s) I. DOI: 10.1107/S1600536813014396/go2090Isup2.hkl

Supplementary material file. DOI: 10.1107/S1600536813014396/go2090Isup3.cml

Additional supplementary materials:  crystallographic information; 3D view; checkCIF report

## Figures and Tables

**Table 1:** Hydrogen-bond geometry (Å, °)

*D*—H⋯*A*	*D*—H	H⋯*A*	*D*⋯*A*	*D*—H⋯*A*
C20—H20⋯O7^i^	0.93	2.50	3.394 (2)	162

Symmetry code: (i) 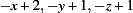.

**Fig. 1.**
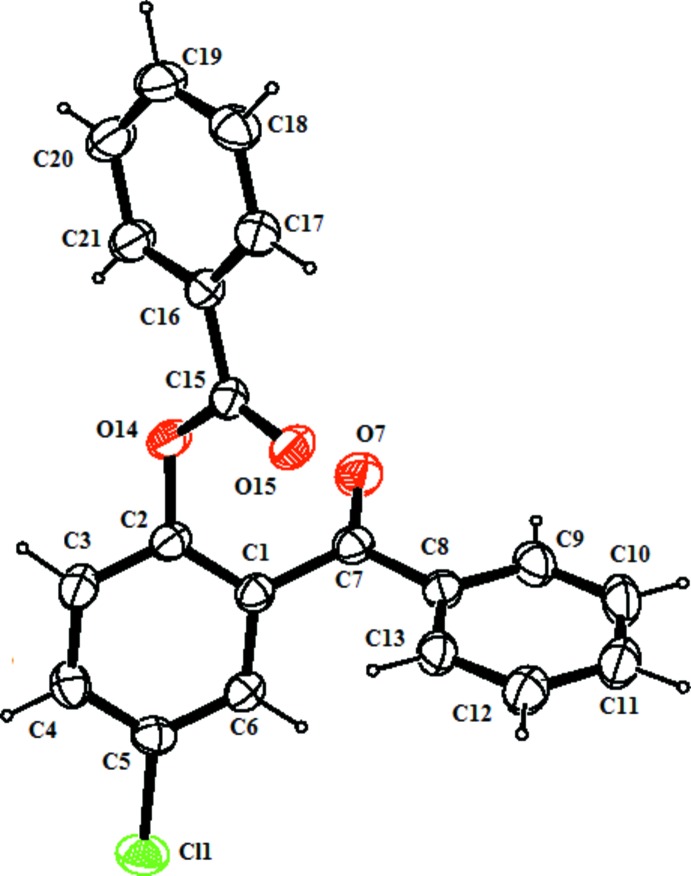
ORTEP view of the molecule with the atom-labelling scheme. The thermal ellipsoids are drawn at the 40% probability level. H atoms are shown as small spheres of arbitrary radii.

**Fig. 2.**
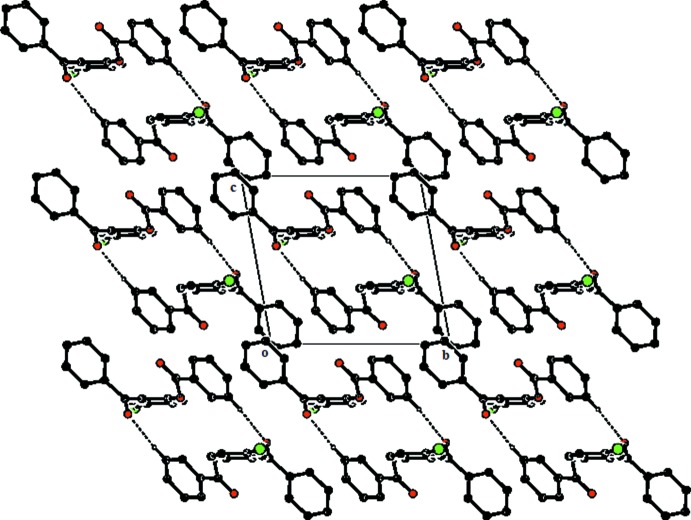
The packing arrangement of molecules viewed down the a axis.

## supplementary crystallographic information

### Comment

The benzophenone nucleus is an important part of the therapeutically interesting drug candidate as inhibitors of HIV-1 reverse transcriptase RT, cancer (Revesz *et al.*, 2004) and inflammatory (Khanum *et al.*, 2004; Khanum *et al.*, 2009; Khanum *et al.*, 2010). Therefore, a number of benzophenone analogues were synthesized, and their chemistry has been extensively studied. The benzophenone moiety, a structural element often seen in compounds from natural sources, presents a variety of biological activities such as anti-inflammatory, antimalarial and anticancer and demonstrated to be a versatile pharmacophoric nucleus, largely used in medicinal chemistry programs (Belluti *et al.*, 2011). The importance of these substances is basically due to the diverse biological and chemical properties that they possess. In view of the above importance and to understand the conformation of the benzophenone moiety, the crystal structure determination of the title compound, was carried out. Bond lengths and bond angles of the title molecule show a fair amount of agreement with some related molecules related structures (Sieroń *et al.*, 2004; Naveen *et al.*, 2006; Mahendra *et al.*, 2005). All bond lengths and angles are within expected values (Allen *et al.*, 1987). The title compound has three benzene rings which are linked by carbonyl and ester groups. The dihedral angles between the ring (C1–C6) and (C8–C13) is 68.82 (5)°, ring (C1–C6) and (C16–C21) is 53.76 (6)° and ring (C8–C13) makes a dihedral angle of 81.17 (5)° with ring (C16–C21). The conformation of attachment of the benzoyl and benzoate rings to the central benzene ring can be characterized by torsion angles C6—C1—C7—C8 and C1—C2—O14—C15 of -54.9 (2) and -58.4 (2)°, respectively. The double bonds C7═O7 and C15═O15 are confirmed by their respective distances of 1.214 (2) and 1.196 (2) Å. Packing view of the molecules in the unit cell viewed down the *a* axis is shown in Fig. 2. The molecules are linked by intermolecular C20—H20···O7 interactions through hydrogen bonding of the carbonyl (benzophenone moiety) and ester substituent. The interaction with a neighbouring molecule is related to the other by a centre of inversion and form hydrogen-bonded dimer unit. Each unit is independently stacked when viewed down the *a* axis Fig.3.

### Experimental

To a solution of (2-hydroxy-5-chlorophenyl) phenyl methanone (1, 1.99 g, 8.6 mmol) in 10% sodium hydroxide solution, benzoyl chloride (1.10 g, 8.6 mmol) was added with constant stirring. The reaction mixture was cooled to 0°C, made alkaline by adding 10% sodium solution and stirring was continued for about 1 h. The separated solid was extracted with ether (3 × 20 ml), the organic layer was washed with 10% sodium hydroxide solution (3 × 15 ml) and with distilled water (3 × 30 ml). The organic layer was dried over anhydrous sodium sulfate and ether was removed to afford crude product, which on recrystallization with alcohol gave white crystals of title compound. Yield: 71%, m.p. 365–367K.

### Refinement

All H atoms were positioned geometrically and were treated as riding on their parent C atoms, with C—H distances of 0.93 Å with *U*_iso_(H) = 1.2*U*_eq_(C).

### Crystal data

**Table:**

C_20_H_13_ClO_3_	*Z* = 2
*M**_r_* = 336.75	*F*(000) = 348
Triclinic, *P*1	*D*_x_ = 1.397 Mg m^−^^3^
Hall symbol: -P 1	Melting point: 367 K
*a* = 9.1934 (2) Å	Mo *K*α radiation, λ = 0.71073 Å
*b* = 9.8641 (3) Å	Cell parameters from 10537 reflections
*c* = 10.0778 (3) Å	θ = 3.5–29.1°
α = 94.033 (2)°	µ = 0.25 mm^−^^1^
β = 114.207 (2)°	*T* = 293 K
γ = 102.512 (2)°	Block, white
*V* = 800.64 (4) Å^3^	0.30 × 0.20 × 0.20 mm

### Data collection

**Table:**

Oxford Xcalibur Sapphire3 diffractometer	3131 independent reflections
Radiation source: fine-focus sealed tube	2631 reflections with *I* > 2σ(*I*)
Graphite monochromator	*R*_int_ = 0.031
Detector resolution: 16.1049 pixels mm^-1^	θ_max_ = 26.0°, θ_min_ = 3.5°
ω scans	*h* = −11→11
Absorption correction: multi-scan (*CrysAlis RED*; Oxford Diffraction, 2010)	*k* = −12→12
*T*_min_ = 0.871, *T*_max_ = 1.000	*l* = −12→12
18813 measured reflections	

### Refinement

**Table:**

Refinement on *F*^2^	Primary atom site location: structure-invariant direct methods
Least-squares matrix: full	Secondary atom site location: difference Fourier map
*R*[*F*^2^ > 2σ(*F*^2^)] = 0.035	Hydrogen site location: inferred from neighbouring sites
*wR*(*F*^2^) = 0.084	H-atom parameters constrained
*S* = 1.03	*w* = 1/[σ^2^(*F*_o_^2^) + (0.0283*P*)^2^ + 0.3095*P*] where *P* = (*F*_o_^2^ + 2*F*_c_^2^)/3
3131 reflections	(Δ/σ)_max_ = 0.001
217 parameters	Δρ_max_ = 0.20 e Å^−^^3^
0 restraints	Δρ_min_ = −0.21 e Å^−^^3^

### Special details

**Table:**

Experimental. *CrysAlis PRO*, Oxford Diffraction Ltd., Version 1.171.34.40 (release 27–08-2010 CrysAlis171. NET) (compiled Aug 27 2010,11:50:40) Empirical absorption correction using spherical harmonics, implemented in SCALE3 ABSPACK scaling algorithm.Elemental analysis for C_20_H_13_ClO_3_: IR (nujol): 1660 (C═O), 1750 cm^-1^ (ester, C═O); ^1^H NMR (CDCl_3_): δ 6.9–7.6 (m, 13H, Ar—H). Analysis, calculated for C_20_H_13_ClO_3_ (336.5): C 71.33, H 3.89, Cl 0.53; found: C 71.39, H 3.72, Cl 10.44%.
Geometry. All e.s.d.'s (except the e.s.d. in the dihedral angle between two l.s. planes) are estimated using the full covariance matrix. The cell e.s.d.'s are taken into account individually in the estimation of e.s.d.'s in distances, angles and torsion angles; correlations between e.s.d.'s in cell parameters are only used when they are defined by crystal symmetry. An approximate (isotropic) treatment of cell e.s.d.'s is used for estimating e.s.d.'s involving l.s. planes.
Refinement. Refinement of *F*^2^ against ALL reflections. The weighted *R*-factor *wR* and goodness of fit *S* are based on *F*^2^, conventional *R*-factors *R* are based on *F*, with *F* set to zero for negative *F*^2^. The threshold expression of *F*^2^ > σ(*F*^2^) is used only for calculating *R*-factors(gt) *etc*. and is not relevant to the choice of reflections for refinement. *R*-factors based on *F*^2^ are statistically about twice as large as those based on *F*, and *R*-factors based on ALL data will be even larger.

### Fractional atomic coordinates and isotropic or equivalent isotropic displacement parameters (Å^2^)

**Table:**

	*x*	*y*	*z*	*U*_iso_*/*U*_eq_	
Cl1	0.13053 (5)	0.16990 (5)	0.62243 (6)	0.05937 (15)	
O14	0.79582 (13)	0.43743 (11)	0.68298 (12)	0.0453 (3)	
O15	0.94425 (14)	0.36025 (11)	0.88776 (12)	0.0459 (3)	
C6	0.43806 (19)	0.16383 (16)	0.66293 (16)	0.0384 (3)	
H6	0.4044	0.0682	0.6644	0.046*	
C2	0.64376 (18)	0.36708 (16)	0.67781 (16)	0.0373 (3)	
C1	0.59678 (18)	0.22283 (15)	0.67650 (16)	0.0357 (3)	
C5	0.33045 (18)	0.24639 (17)	0.64737 (17)	0.0400 (4)	
C7	0.70368 (18)	0.12708 (15)	0.67663 (17)	0.0385 (3)	
C15	0.94012 (18)	0.43373 (15)	0.79760 (16)	0.0356 (3)	
C16	1.08476 (18)	0.53267 (15)	0.79432 (17)	0.0355 (3)	
O7	0.75790 (16)	0.12731 (13)	0.58523 (15)	0.0569 (3)	
C8	0.73479 (18)	0.03063 (15)	0.78589 (17)	0.0376 (3)	
C3	0.5371 (2)	0.44936 (16)	0.66627 (18)	0.0439 (4)	
H3	0.5719	0.5459	0.6696	0.053*	
C21	1.0687 (2)	0.61313 (17)	0.68447 (19)	0.0466 (4)	
H21	0.9646	0.6049	0.6085	0.056*	
C4	0.3790 (2)	0.38951 (17)	0.64981 (18)	0.0438 (4)	
H4	0.3063	0.4447	0.6405	0.053*	
C13	0.71872 (19)	0.05701 (16)	0.91536 (18)	0.0422 (4)	
H13	0.6800	0.1331	0.9321	0.051*	
C17	1.24005 (19)	0.54643 (16)	0.90776 (18)	0.0417 (4)	
H17	1.2518	0.4925	0.9817	0.050*	
C18	1.3775 (2)	0.64032 (18)	0.9110 (2)	0.0496 (4)	
H18	1.4816	0.6503	0.9877	0.060*	
C9	0.7918 (2)	−0.08432 (17)	0.7620 (2)	0.0481 (4)	
H9	0.8034	−0.1032	0.6757	0.058*	
C19	1.3604 (2)	0.71918 (18)	0.8006 (2)	0.0527 (4)	
H19	1.4531	0.7818	0.8026	0.063*	
C20	1.2068 (2)	0.70554 (19)	0.6876 (2)	0.0553 (5)	
H20	1.1958	0.7586	0.6131	0.066*	
C11	0.8161 (2)	−0.14242 (18)	0.9953 (2)	0.0539 (5)	
H11	0.8441	−0.2001	1.0659	0.065*	
C12	0.7599 (2)	−0.02918 (18)	1.0198 (2)	0.0505 (4)	
H12	0.7495	−0.0105	1.1068	0.061*	
C10	0.8310 (2)	−0.17028 (17)	0.8670 (2)	0.0551 (5)	
H10	0.8678	−0.2476	0.8504	0.066*	

### Atomic displacement parameters (Å^2^)

**Table:**

	*U*^11^	*U*^22^	*U*^33^	*U*^12^	*U*^13^	*U*^23^
Cl1	0.0354 (2)	0.0713 (3)	0.0687 (3)	0.0086 (2)	0.0220 (2)	0.0177 (2)
O7	0.0698 (9)	0.0553 (7)	0.0646 (8)	0.0210 (6)	0.0445 (7)	0.0177 (6)
O14	0.0337 (6)	0.0453 (6)	0.0512 (7)	0.0043 (5)	0.0140 (5)	0.0220 (5)
O15	0.0434 (6)	0.0429 (6)	0.0474 (7)	0.0067 (5)	0.0168 (5)	0.0174 (5)
C1	0.0348 (8)	0.0362 (8)	0.0338 (8)	0.0081 (6)	0.0132 (6)	0.0089 (6)
C2	0.0327 (8)	0.0382 (8)	0.0366 (8)	0.0055 (6)	0.0121 (6)	0.0121 (6)
C3	0.0429 (9)	0.0341 (8)	0.0464 (9)	0.0090 (7)	0.0115 (7)	0.0107 (7)
C4	0.0402 (9)	0.0443 (9)	0.0437 (9)	0.0166 (7)	0.0127 (7)	0.0078 (7)
C5	0.0310 (8)	0.0484 (9)	0.0360 (8)	0.0073 (7)	0.0119 (7)	0.0074 (7)
C6	0.0385 (8)	0.0349 (8)	0.0375 (8)	0.0054 (6)	0.0143 (7)	0.0078 (6)
C7	0.0348 (8)	0.0343 (8)	0.0429 (9)	0.0039 (6)	0.0167 (7)	0.0043 (6)
C8	0.0308 (8)	0.0310 (7)	0.0463 (9)	0.0058 (6)	0.0137 (7)	0.0054 (6)
C9	0.0436 (9)	0.0396 (9)	0.0581 (11)	0.0116 (7)	0.0202 (8)	0.0032 (8)
C10	0.0467 (10)	0.0340 (8)	0.0779 (14)	0.0161 (8)	0.0182 (10)	0.0093 (8)
C11	0.0463 (10)	0.0418 (9)	0.0638 (12)	0.0107 (8)	0.0134 (9)	0.0201 (8)
C12	0.0515 (10)	0.0476 (10)	0.0500 (10)	0.0127 (8)	0.0192 (8)	0.0150 (8)
C13	0.0427 (9)	0.0345 (8)	0.0487 (9)	0.0116 (7)	0.0183 (8)	0.0082 (7)
C15	0.0375 (8)	0.0307 (7)	0.0375 (8)	0.0091 (6)	0.0152 (7)	0.0067 (6)
C16	0.0353 (8)	0.0313 (7)	0.0387 (8)	0.0071 (6)	0.0162 (7)	0.0049 (6)
C17	0.0409 (9)	0.0409 (8)	0.0403 (9)	0.0103 (7)	0.0153 (7)	0.0066 (7)
C18	0.0338 (9)	0.0498 (10)	0.0534 (10)	0.0048 (7)	0.0124 (8)	−0.0009 (8)
C19	0.0426 (10)	0.0439 (9)	0.0683 (12)	−0.0011 (7)	0.0281 (9)	0.0052 (8)
C20	0.0533 (11)	0.0502 (10)	0.0635 (12)	0.0068 (8)	0.0280 (10)	0.0241 (9)
C21	0.0389 (9)	0.0460 (9)	0.0497 (10)	0.0065 (7)	0.0156 (8)	0.0169 (8)

### Geometric parameters (Å, º)

**Table:**

C1—C2	1.392 (2)	C11—C12	1.375 (2)
C1—C6	1.394 (2)	C11—H11	0.9300
C1—C7	1.503 (2)	C12—C13	1.383 (2)
C2—C3	1.378 (2)	C12—H12	0.9300
C2—O14	1.3977 (17)	C13—H13	0.9300
C3—C4	1.381 (2)	C15—O15	1.1952 (17)
C3—H3	0.9300	C15—O14	1.3660 (18)
C4—C5	1.380 (2)	C15—C16	1.483 (2)
C4—H4	0.9300	C16—C21	1.385 (2)
C5—C6	1.381 (2)	C16—C17	1.385 (2)
C5—Cl1	1.7333 (15)	C17—C18	1.382 (2)
C6—H6	0.9300	C17—H17	0.9300
C7—O7	1.2138 (19)	C18—C19	1.378 (3)
C7—C8	1.485 (2)	C18—H18	0.9300
C8—C13	1.385 (2)	C19—C20	1.373 (3)
C8—C9	1.393 (2)	C19—H19	0.9300
C9—C10	1.384 (2)	C20—C21	1.381 (2)
C9—H9	0.9300	C20—H20	0.9300
C10—C11	1.370 (3)	C21—H21	0.9300
C10—H10	0.9300		
			
C2—C1—C6	117.95 (14)	C10—C11—H11	120.0
C2—C1—C7	122.98 (13)	C12—C11—H11	120.0
C6—C1—C7	118.88 (13)	C11—C12—C13	120.16 (17)
C3—C2—C1	121.19 (14)	C11—C12—H12	119.9
C3—C2—O14	115.25 (13)	C13—C12—H12	119.9
C1—C2—O14	123.49 (14)	C12—C13—C8	120.34 (15)
C2—C3—C4	120.46 (14)	C12—C13—H13	119.8
C2—C3—H3	119.8	C8—C13—H13	119.8
C4—C3—H3	119.8	O15—C15—O14	122.79 (13)
C5—C4—C3	118.93 (15)	O15—C15—C16	126.12 (14)
C5—C4—H4	120.5	O14—C15—C16	111.09 (12)
C3—C4—H4	120.5	C21—C16—C17	119.55 (14)
C4—C5—C6	120.98 (14)	C21—C16—C15	122.27 (14)
C4—C5—Cl1	119.15 (12)	C17—C16—C15	118.16 (13)
C6—C5—Cl1	119.87 (12)	C18—C17—C16	120.00 (15)
C5—C6—C1	120.46 (14)	C18—C17—H17	120.0
C5—C6—H6	119.8	C16—C17—H17	120.0
C1—C6—H6	119.8	C19—C18—C17	120.01 (16)
O7—C7—C8	121.80 (14)	C19—C18—H18	120.0
O7—C7—C1	119.47 (14)	C17—C18—H18	120.0
C8—C7—C1	118.69 (13)	C20—C19—C18	120.21 (15)
C13—C8—C9	119.11 (15)	C20—C19—H19	119.9
C13—C8—C7	121.70 (14)	C18—C19—H19	119.9
C9—C8—C7	119.07 (15)	C19—C20—C21	120.11 (16)
C10—C9—C8	119.83 (17)	C19—C20—H20	119.9
C10—C9—H9	120.1	C21—C20—H20	119.9
C8—C9—H9	120.1	C20—C21—C16	120.11 (15)
C11—C10—C9	120.57 (16)	C20—C21—H21	119.9
C11—C10—H10	119.7	C16—C21—H21	119.9
C9—C10—H10	119.7	C15—O14—C2	119.99 (11)
C10—C11—C12	119.99 (16)		
			
C6—C1—C2—C3	0.1 (2)	C8—C9—C10—C11	−0.8 (3)
C7—C1—C2—C3	175.00 (14)	C9—C10—C11—C12	0.8 (3)
C6—C1—C2—O14	−176.52 (13)	C10—C11—C12—C13	−0.2 (3)
C7—C1—C2—O14	−1.6 (2)	C11—C12—C13—C8	−0.4 (3)
C1—C2—C3—C4	−1.4 (2)	C9—C8—C13—C12	0.5 (2)
O14—C2—C3—C4	175.44 (14)	C7—C8—C13—C12	−175.37 (15)
C2—C3—C4—C5	1.0 (2)	O15—C15—C16—C21	179.39 (16)
C3—C4—C5—C6	0.8 (2)	O14—C15—C16—C21	−1.5 (2)
C3—C4—C5—Cl1	−178.51 (13)	O15—C15—C16—C17	−2.2 (2)
C4—C5—C6—C1	−2.2 (2)	O14—C15—C16—C17	176.90 (13)
Cl1—C5—C6—C1	177.15 (12)	C21—C16—C17—C18	0.2 (2)
C2—C1—C6—C5	1.7 (2)	C15—C16—C17—C18	−178.25 (14)
C7—C1—C6—C5	−173.43 (14)	C16—C17—C18—C19	−0.7 (3)
C2—C1—C7—O7	−52.1 (2)	C17—C18—C19—C20	0.4 (3)
C6—C1—C7—O7	122.75 (17)	C18—C19—C20—C21	0.3 (3)
C2—C1—C7—C8	130.12 (15)	C19—C20—C21—C16	−0.8 (3)
C6—C1—C7—C8	−55.02 (19)	C17—C16—C21—C20	0.5 (3)
O7—C7—C8—C13	160.10 (16)	C15—C16—C21—C20	178.90 (16)
C1—C7—C8—C13	−22.2 (2)	O15—C15—O14—C2	8.1 (2)
O7—C7—C8—C9	−15.7 (2)	C16—C15—O14—C2	−171.02 (13)
C1—C7—C8—C9	161.98 (14)	C3—C2—O14—C15	124.88 (15)
C13—C8—C9—C10	0.1 (2)	C1—C2—O14—C15	−58.3 (2)
C7—C8—C9—C10	176.07 (15)		

### Hydrogen-bond geometry (Å, º)

**Table:**

*D*—H···*A*	*D*—H	H···*A*	*D*···*A*	*D*—H···*A*
C20—H20···O7^i^	0.93	2.50	3.394 (2)	162

Symmetry code: (i) −*x*+2, −*y*+1, −*z*+1.
